# Definition of RING gene family in sugar beet (*Beta vulgaris* L.) and functional characterization of *BvRING-HCa-9* in plant salt stress tolerance

**DOI:** 10.1186/s12870-026-09086-5

**Published:** 2026-07-25

**Authors:** Xinyu Jiang, Shuang Wang, Yu Bing, Chunquan Ma, Sixue Chen, Yuguang Wang, Haiying Li

**Affiliations:** 1 Engineering Research Center of Agricultural Microbiology Technology, Ministry of Education and Heilongjiang Provincial Key Laboratory of Plant Genetic Engineering and Biological Fermentation Engineering for Cold Region and Key Laboratory of Molecular Biology, College of Heilongjiang Province and School of Life Sciences, Heilongjiang University, Harbin, Heilongjiang 150080 P.R. China; 2 Department of Biology, University of Mississippi, Oxford, MS USA; 3 Heilongjiang University, Harbin, Heilongjiang 150080 China

**Keywords:** Sugar beet, RING family genes, Salt tolerance, *Bv*RING-HCa-9

## Abstract

**Background:**

RING-type E3 ligases are key enzymes in protein ubiquitination modification and crucial regulators in plant growth and development. However, their roles in sugar beet (*Beta vulgaris* L.) remain unclear. To determine the types and distribution of RING-type proteins in sugar beet, we conducted a comprehensive analysis of the RING family genes. By analyzing *RING* gene expression profiles under salt stress, a candidate gene (*BvRING-HCa-9*) associated with salt stress was identified. The goal is to characterize the functions of the sugar beet RING gene family in plant salt stress tolerance.

**Results:**

A total of 446 RING-type proteins in the sugar beet genome were identified. The coding genes are distributed across all nine chromosomes, with varying expression patterns among subtypes. Collinearity and Ka/Ks analyses suggest that the RING family in sugar beet has undergone limited duplication events and is under strong purifying selection, indicating evolutionary conservation. Many *BvRING* genes are transcriptionally responsive to salt stress. Among them, *Bv*RING-HCa-9 possesses ubiquitin E3 ligase activity and is localized in the nucleus and plasma membrane. Overexpression of *BvRING-HCa-9* in transgenic Arabidopsis enhanced salt tolerance.

**Conclusion:**

The 446 RING-domain genes in sugar beet are evolutionarily conserved, and majority of the genes are transcriptionally responsive to salt stress. Among them, *BvRING-HCa-9* was characterized to encode a E3 ligase and it plays an important role in plant salt tolerance. These findings provide a valuable resource for functional studies of other RING family genes in plant stress resilience.

**Supplementary Information:**

The online version contains supplementary material available at https://doi.org/10.1186/s12870-026-09086-5.

## Background

Ubiquitination is a type of protein post-translational modification (PTM), where ubiquitin molecules are covalently attached to target proteins through an enzymatic cascade involving E1 ubiquitin-activating enzyme, E2 ubiquitin-conjugating enzymes, and E3 ubiquitin ligases [1, 2]. Depending on the chain length and linkage type, ubiquitination can lead to target protein degradation either by the proteasome or via the vacuolar/lysosomal pathway, or changes in subcellular localization and signaling functions [3, 4]. The E3 ubiquitin ligases specifically recognize substrate proteins and facilitate the transfer of ubiquitin molecules to the substrate, making them key enzymes in the ubiquitination reaction [5, 6]. RING (Really Interesting New Gene) type proteins constitue the largest subfamily of E3 ubiquitin ligases. They are characterized by a conserved RING domain consisting of 40–60 amino acids, which forms a unique cross-brace structure by coordinating two zinc ions, thereby mediating ubiquitination modification of target proteins [7]. Identification and functional analysis of the RING-type protein family have progressed in multiple plants. A total of 469 RING family members were identified in the Arabidopsis genome and classified into eight groups [8]. Similarly, 590 RING family members were identified in the maize genome and classified into six groups. A drought-related member *ZmRING-93* was identified, and heterologous overexpression of *ZmRING-93* showed a clear drought tolerance function [9]. Additionally, 514, 509, and 914 RING genes were identified in *Gossypium arboreum*, *G. raimondii*, and *G. hirsutum*, respectively, among which overexpression of a *GhZFRG1* gene in Arabidopsis enhanced plant tolerance to salt stress [10].

RING-type family members are widely involved in core biological processes, such as growth and development, hormone signal transduction, and responses to biotic and abiotic stresses [11, 12]. The RING-type protein TEAR4 (TIE1-Associated RING-Type E3 Ligase 4) in Arabidopsis functions by mediating the degradation of AtRGA (Repressor of Ga1-3) and AtGAI (Gibberellic Acid Insensitive) in the DELLA family to regulate Arabidopsis seed germination [13]. In the classic auxin signaling pathway, TIR1 (Transport Inhibitor Response 1) serves as an auxin receptor and possesses RING-type E3 ubiquitin ligase activity. TIR1 can promote the ubiquitination and degradation of Aux/IAA (Auxin/Indole-3-Acetic Acid proteins) through its E3 ligase activity, relieving their repression on ARF (Auxin Response Factor) transcription factors, playing a significant role in auxin signal regulation [14]. The RING-type E3 ubiquitin ligase *Os*RMT1 (RING-type E3 ligase for Multiple Stress Tolerance 1) is a negative regulator of salt tolerance in rice. It physically interacts with the positive salt-responsive regulator *Os*MBL1 (Mannose-Binding Lectin 1) to mediate ubiquitination and degradation of *Os*MBL1. Under salt stress, the transcriptional level of *OsRMT1* decreases, weakening its degradation of *Os*MBL1. The accumulated *Os*MBL1 binds to *Os*ERF040 (Ethylene Responsive Factor 040), thereby activating downstream salt tolerance gene expression and enhancing rice salt tolerance [15]. All members of the rice SINARs (SINA‑like RING E3 ligases) contain conserved RING domains and can ubiquitinate *Os*ER1 (ERECTA 1), but their functions are antagonistic. SINAR2-SINAR5 can mediate *Os*ER1 ubiquitination and degradation, positively and redundantly regulating grains per panicle; while SINAR1 and SINAR6 can form heterodimers, inhibiting the former’s modification and degradation of *Os*ER1. In the SINAR1 and SINAR6 double mutant, *Os*ER1 abundance decreases, and rice grain number per panicle increased [16]. In summary, the diverse functions of RING-type proteins in plant growth and stress responses make them core research targets in crop breeding [17, 18].

Sugar beet (*Beta vulgaris* L.) possesses salt-tolerant characteristics. It is rich in osmolytes such as betaine and soluble sugars, which function synergistically to maintain cellular osmotic balance. They help to maintain growth and metabolic activity under high-salt environments, making sugar beet a good model for studying crop salt tolerance mechanisms [19, 20]. Sugar beet is also one of China’s major sugar crops with high economic value [21]. Although RING-type proteins are well known to be involved in plant growth and stress responses, they have not been explored in sugar beet. Therefore, identifying the RING family members with functions in the salt-tolerant and economically valuable sugar beet is of great significance.

This study identified RING family members in the sugar beet genome using RING-type conserved domain models, aiming to comprehensively analyze the molecular characteristics and evolutionary mechanisms of the sugar beet RING-type protein family and screen candidate genes responsive to salt stress. A total of 446 RING-type proteins were identified and one of the genes, *BvRING-HCa-9*, showing significant expression changes in response to salt stress. *Bv*RING-HCa-9 possesses ubiquitination activity and its overexpression in *Arabidopsis thaliana* enhanced plant salt stress tolerance. This study fills the gap in RING family identification in the non-model crop sugar beet, provides an important reference for RING gene characterization in salt stress response and for molecular breeding of crop salt tolerance.

## Materials and methods

### Screening and classification of sugar beet RING-type proteins

To obtain all proteins containing the RING conserved domain in sugar beet, we downloaded HMM (Hidden Markov Model) conserved domain models containing RING (PF00097, PF13920, PF13923, PF13639, PF15227, PF12906) from InterPro (https://www.ebi.ac.uk/interpro/) [20]. Using the hmmsearch tool, we screened for RING-type protein members in the sugar beet genome. Sugar beet protein and genome sequences were downloaded from the NCBI website (https://www.ncbi.nlm.nih.gov/) [22], with the sugar beet reference genome being EL 10.2. The initially obtained sugar beet protein sequences containing RING domains were further screened using the CD Search on the NCBI website and the Batch access module on the SMART website (https://smart.embl.de/) [23], with E-value < 0.01. Based on the positional characteristics of the eight conserved amino acids in the obtained protein conserved domains, they were classified into multiple types and annotated accordingly.

### Chromosomal localization and collinearity analysis

Sugar beet genome nucleic acid sequences and chromosome sequence numbers were obtained from NCBI. Tbtools was used to extract chromosomal localization information of *Bv*RING-type protein-coding genes and visualize it combined with sugar beet chromosome density information [24]. We also used the One Step MCScanX module of Tbtools to analyze the evolutionary relationships of RING-type proteins within sugar beet and with close relatives, distant relatives, and model plants (spinach, rice, and Arabidopsis) and visualized them. The Simple Ka/Ks Calculator [25] was used to calculate the evolutionary selection pressure on protein-coding genes.

### Phylogenetic tree construction

MEGA11 software was used to perform multiple sequence alignment (ClustalW) and construct a phylogenetic tree (Maximum likelihood method) using the amino acid sequences of all the identified proteins. The test method for the phylogenetic tree was the Bootstrap method [26], with parameters set to 1000 replicates. The resulting tree was beautified using iTOL (https://itol.embl.de/) [27].

### Promoter *cis*-acting element and conserved domain analysis

The CD Search on the NCBI website was used to analyze conserved domains contained in the amino acid sequences of RING-type proteins, and visualized in combination with the phylogenetic tree (https://www.ncbi.nlm.nih.gov/Structure/bwrpsb/bwrpsb.cgi/). Tbtools was used to extract the 2000 bp upstream sequences of sugar beet RING protein-coding genes. Promoter element composition was analyzed on the plantCARE software (https://bioinformatics.psb.ugent.be/webtools/plantcare/html/) [28], and data were organized to draw a heatmap.

### Analysis of RING gene expression trends under salt stress

RNA-seq data of sugar beet leaves and roots under salt stress were downloaded from the NCBI SRA database (Accession: PRJNA666117). Expression data for sugar beet RING genes were filtered. The meta-analysis online platform (https://analysis.obiosh.com) was used to analyze the salt-responsive expression trends of RING genes in sugar beet roots and leaves and plot the results [29].

### In vitro ubiquitination assay

The coding sequences of *BvRING-HCa-9* (E3), *UBCH5b* (E2), and *UBQ14* (ubiquitin) were cloned into a pET-28a vector containing a His tag. HIS (pET-28a) was used as a negative control. The contructs were transformed into BL21 (DE3) strains to induce protein expression. The protein was purified using a Nickel column system as described in manufacturer manual (C600332, Sangon). The reaction included UBA (UBE1, UB-biotech), the purified *Bv*RING-HCa-9 (E3), UBCH5b (E2), and UBQ14 (UBQ) in a reaction buffer (250 mmol/L TrisHCl, 12.5 mmol/L MgCl_2_, 2.5 mmol/L DTT, 10 mmol/L ATP) at 28 °C for 4 h. The protein samples were boiled and dissolved in SDS-PAGE gel. Western blot was performed with anti-HIS antibody (D410002, Sangon) and an anti-ubiquitin antibody (D291607, Sangon) was used at a dilution of 1:1000 with a TBST solution (20 mM Tris, 150 mM NaCl, 0.1%Tween-20, pH为7.4).

### Subcellular localization analysis

The coding region (CDS) of the *BvRING-HCa-9* gene was cloned into the pCAMBIA2300-eYFP vector. The constructed recombinant vector or empty vector (pCAMBIA2300-eYFP) was transformed into tobacco leaves via Agrobacterium-mediated transformation and cultured for 24 h (after 24 h of dark culture). Laser confocal microscopy was used to observe the distribution of fluorescent signals from the fusion protein (FV1200, Olympus, Tokyo, Japan).

### Plant cultivation and transgenic plant acquisition

Sugar beet seeds (T710MU) were germinated in vermiculite. Germinated seedlings were transplanted into 1/2 Hoagland nutrient solution and cultured in a growth chamber (16-hour light / 8-hour dark cycle, 50% relative humidity). *A. thaliana* Columbia ecotype (Columbia-0, Col-0) and tobacco (*Nicotiana benthamiana*) seeds were vernalized at 4 °C for 1 day, sown in a vermiculite: soil = 1:1 mixture, cultured in the dark for 1 day, then transferred to a growth chamber for continued cultivation. Arabidopsis (Col-0) seeds were obtained from the ABRC (abrc.org). Sugar beet (T710MU) and tobacco (*Nicotiana benthamiana*) seeds are the property of the laboratory.

Arabidopsis overexpression (OE) transgenic plants were obtained using the Agrobacterium-mediated floral dip method [28]. Positive OE plants were screened using Hygromycin B (30 mg/L). After four weeks of growth, plant leaf DNA and RNA were extracted for DNA verification and RT-PCR analysis, respectively. Ultimately, we generated three overexpression lines (OE#1, OE#2, OE#3).

### Physiological indicator detection

When plants grew to the stage with several pairs of true leaves, Arabidopsis plants with consistent growth status were selected for 150 mM NaCl salt stress treatment, with WT Arabidopsis as the control and OE lines 1, 2, 3 as the experimental group. Samples were taken when seedling leaves showed curling or yellowing phenotypes. Total chlorophyll content was measured using the alcohol extraction method, with the calculation formula: C (mg/g) = A652 × 1000 × V / (34.5 × W). Root length and germination rate of transgenic and wild-type seedlings under stress treatment (including 150 mM NaCl) and non-stress treatment conditions were measured. Dry weight (DW) and fresh weight (FW) of Arabidopsis under 150 mM NaCl stress treatment and non-stress treatment were measured following previously reported methods [29]. Betaine content (Cat. No. TCJ-2-G, Grace Biotechnology Co., Ltd., Suzhou, China), MDA content (Cat. No. MDA-2-Y, Grace Biotechnology Co., Ltd., Suzhou, China), SOD activity (Cat. No. SOD-2-W, Grace Biotechnology Co., Ltd., Suzhou, China), and POD activity (Cat. No. POD-2-Y, Grace Biotechnology Co., Ltd., Suzhou, China) were measured using a commercial kit. E3 ubiquitin ligase activity was measured using a commercial kit (Cat. No. YJ000096, Shanghai Enzyme-linked Biotechnology Co., Ltd., Shanghai, China); Na⁺ and potassium ion (K⁺) content were analyzed by flame atomic absorption spectroscopy.

### Total RNA extraction and quantitative real-time PCR (qRT-PCR) analysis

Total RNA was extracted using the TRIzol method [30]. The TAKARA reverse transcription kit from Takara Biomedical Technology (Beijing) Co., Ltd., China, was used to synthesize qRT-PCR templates. Specific primers were designed using the Primer 3 plus online tool (https://www.primer3plus.com), with *18 S rRNA* as the internal reference gene. qRT-PCR detection was performed using a QuantStudio1 real-time PCR instrument (Thermo Fisher Scientific Inc., USA). Actin and 18 S rRNA were used as internal reference genes, and the 2^−ΔΔCT^ method was used to analyze relative gene expression [31].

## Results

### Identification of RING-type proteins in sugar beet and statistics of conserved metal ligand spacing

In this study, we identified 446 RING-type proteins by comparing sugar beet protein sequences with the RING-type conserved domain model using InterProScan. Based on the position and composition of the eight conserved amino acids (metal ligands) in the RING-type conserved domain, they were classified into nine categories, i.e., RING-H2, RING-E, RING-HCa, RING-HCb, RING-R, RING-D, RING-G, RING-v, and RING-C2. Among them, RING-H2 and RING-HC (including RING-HCa and RING-HCb) types account for 48.7% and 39% respectively, followed by RING-v at 8.1%, while the other six types each account for about 1% (Appendix Table 1) (Table 1).

**Table 1:** Classification and counting of conserved sites of RING family proteins in sugar beet

ml pairs		ml1-X_*n*_-ml2		ml3-X_*n*_-ml4				ml4-X_*n*_-ml5				ml5-X_*n*_-ml6		ml7-X_*n*_-ml8		
No. of amino acid (*n*)		2	3	0	1	2	3	2	3	4	7	2	3	1	2	4
No. of RING domains	RING-H2	216	1	0	216	0	2	216	0	0	0	216	0	2	214	0
	RING-HCa	155	0	0	154	0	1	117	38	0	0	153	2	0	155	0
	RING-HCb	19	0	0	19	0	0	19	0	0	0	19	0	0	0	19
	RING-C2	5	0	3	2	0	0	0	0	3	0	3	0	0	3	0
	RING-v	36	0	0	32	4	0	4	0	3	29	36	0	0	36	0
	RING-D	6	0	0	6	0	0	6	0	0	0	6	0	0	6	0
	RING-G	2	0	0	2	0	0	2	0	0	0	2	0	0	2	0
	RING-E	1	0	0	1	0	0	1	0	0	0	1	0	0	1	0
	RING-R	5	0	0	5	0	0	5	0	0	0	5	0	0	5	0

We counted the number of amino acids between adjacent metal ligands and found that among the seven pairs of metal ligands, the amino acid numbers between the ml2-ml3 and ml7-ml8 pairs varied greatly (Appendix Tables 2 and 3), while the other five pairs had relatively small variations and were more conserved. Among the nine categories of sugar beet RING-type proteins, only one member of the RING-H2 category had 3 amino acids between ml1-ml2, while all the rest had 2. The number of amino acids between the ml3-ml4 ligands was mostly 1, with a few being 2 or 3. Notably, RING-C2 had cases with 0 amino acids between ml3-ml4 ligands. The number of amino acids between ml4-ml5 ligands mainly clustered around 2/3/4, but for RING-v, it was mainly 7. The number of amino acids between ml5-ml6 ligands was consistently 2, except for a few RING-HCa types with 3. The number of amino acids between ml7-ml8 ligands was either 2 or 4, which mainly distinguishes the sugar beet RING-HCa and RING-HCb types; in the RING-HC structure, the latter has 4 amino acids between the ml7-ml8 ligands. For the variable regions, the amino acid numbers for ml2-ml3 and ml7-ml8 in all the types of sugar beet RING proteins are shown in Supplementary Tables 2 and 3, and the spatial distribution of the tertiary structure and conserved sites for one example protein from each category is shown in Supplementary Fig. 1.

**Fig. 1:**
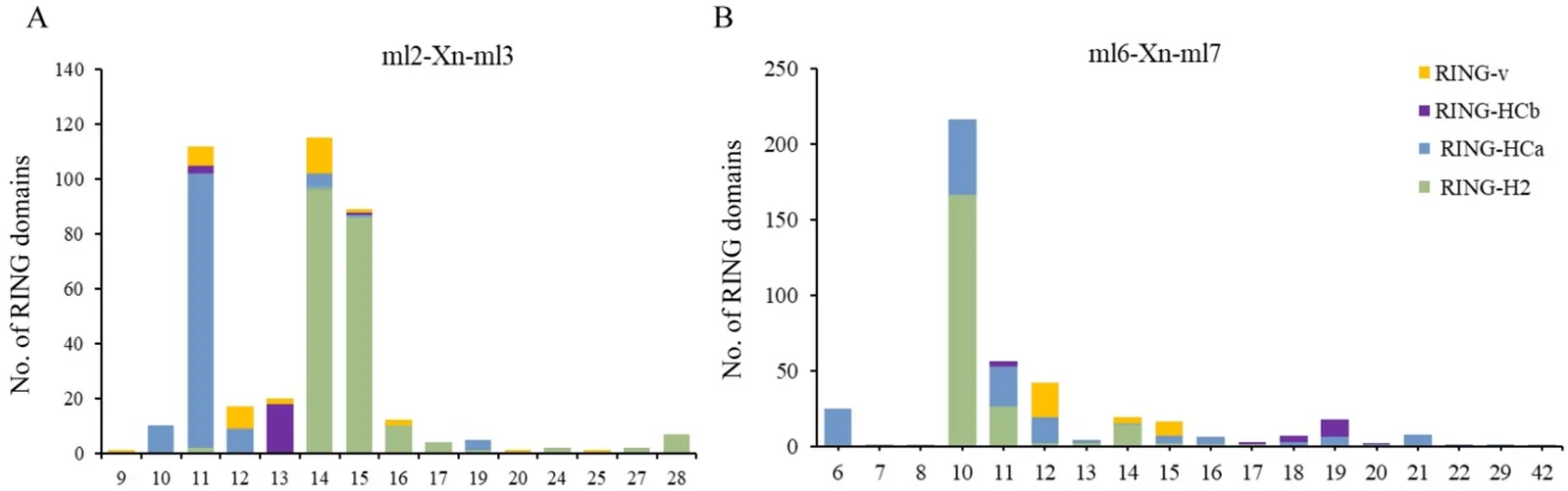
Comparison of RING domain numbers between metal ligand ML2-ML3 and metal ligand ML6-ML7 in sugar beet. **A** metal ligands ML2-ML3; **B** metal ligands ML6-ML7

Furthermore, we displayed the amino acid numbers for the ml2-ml3 and ml6-ml7 pairs in the RING-H2, RING-v, RING-HCa, and RING-HCb categories (Fig. 1). For RING-H2, amino acids between ml2-ml3 ranged from 11 to 28, but mainly clustered at 14 (97/217) and 15 (86/217); amino acids between ml6-ml7 ranged from 7 to 42 (42 being an extreme minority, only 1 member), but mainly clustered at 10 (166/217), 11 (26/217), and 14 (14/217). For RING-HCa, the amino acid count range between ml2-ml3 ligands was 10–19, and between ml6-ml7 ligands was 6–29, with both pairs concentrating at 11 amino acids, the former proportion being (103/155), the latter (53/155). For RING-HCb, the amino acids between ml2-ml3 and ml6-ml7 ligands were concentrated at 13 amino acids (15/19) and 19 amino acids (most members, 9/19) respectively. For RING-v, the amino acid count range between ml2-ml3 ligands was larger, 9–25, while between ml6-ml7 ligands it was mainly concentrated at 12 amino acids (23/36). These results indicate that the distances between adjacent metal ligands in the RING proteins are highly conserved in the core regions (e.g., ml5–ml6), whereas the distances exhibit significant subtype-specific differences in the variable regions (e.g., ml2–ml3 and ml7–ml8).

### Chromosomal localization of sugar beet RING-type proteins

**Fig. 2:**
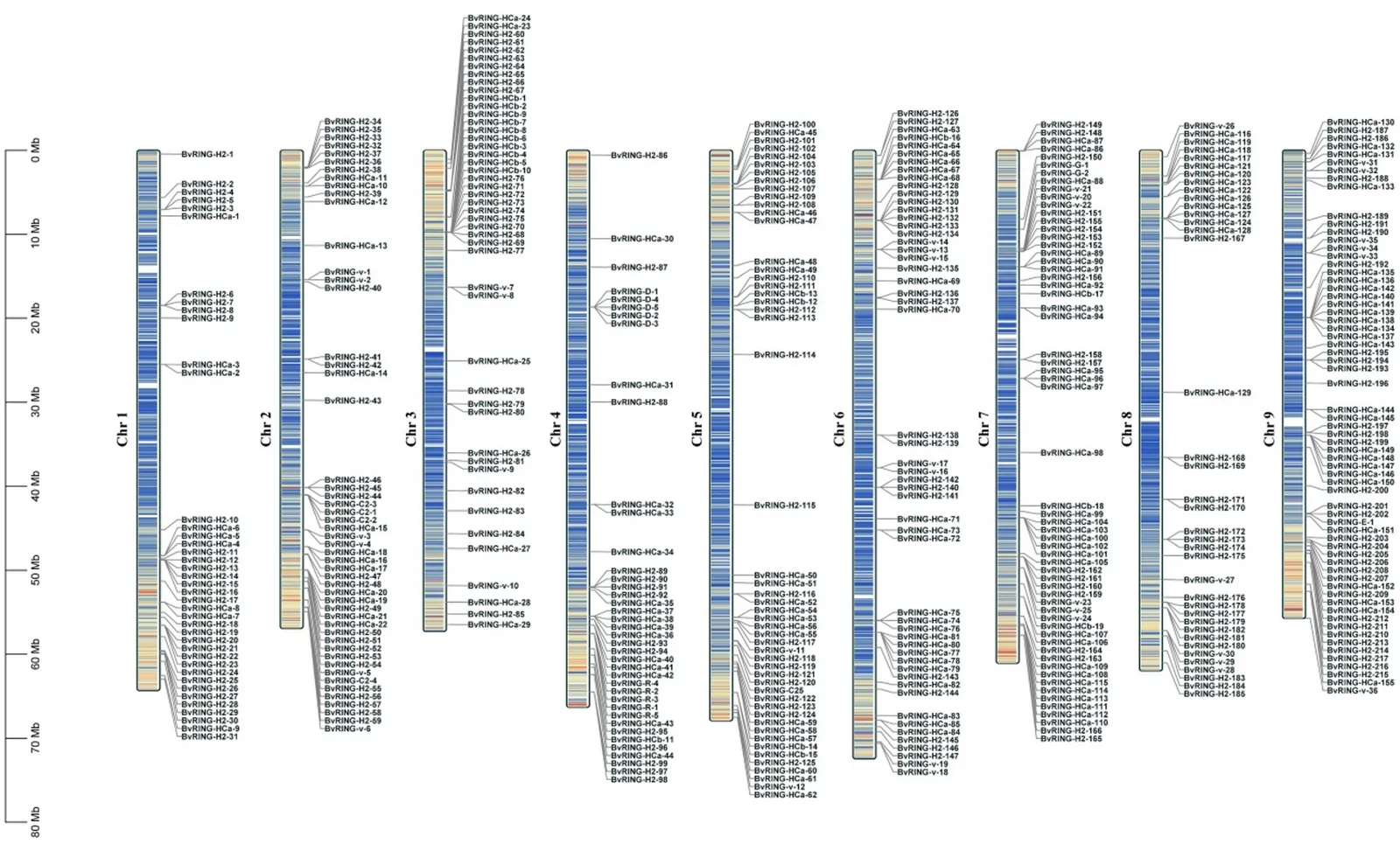
The distribution of 446 *RING* genes on the nine chromosomes of sugar beet. The physical position (Mb) of chromosome is indicated on the left side of figure. Chromosome number (Chr 1 - Chr 9) is indicated at the left of each chromosome. The Gene name is indicated on the right side of each chromosome

Using the amino acid sequence information of all sugar beet RING-type proteins and sugar beet chromosome information, the distribution of the 446 RING-type proteins across the nine sugar beet chromosomes was mapped (Fig. 2). Results show that sugar beet RING-H2 and RING-HCa type members are distributed on all the chromosomes, while sugar beet RING-v members are absent only from Chr1; RING-HCb is distributed on Chr3-9; RING-C2 is distributed on Chr2/Chr5. The categories with fewer members, RING-D, RING-E, RING-R, and RING-G, are distributed on Chr 4, Chr 9, Chr 4, and Chr 7, respectively. Overall, RING-H2 and RING-HCa members are widely distributed across all chromosomes, whereas other subtypes exhibit a biased distribution pattern.

### Collinearity analysis of sugar beet RING-type proteins

**Fig. 3:**
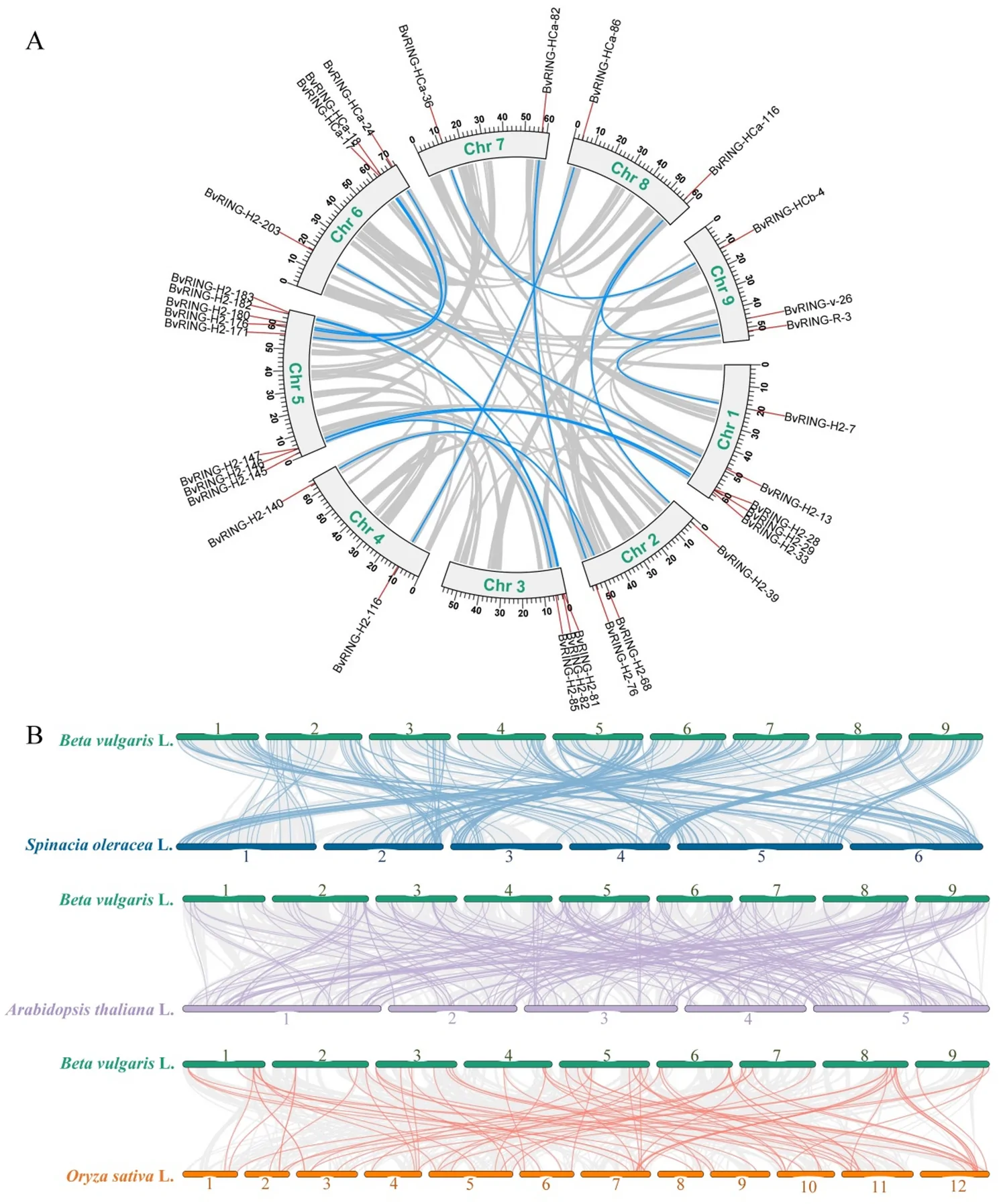
Collinearity analysis of sugar beet with other species. **A** Duplication events of the *Bv*RING gene family in sugar beet. The blue line represents the collinear pairs of the *BvRING* genes, the end of the red line is the gene names, and the chromosome number is indicated as Chr 1–9; **B** The *RING* gene synthesis relationships between sugar beet and spinach, sugar beet and Arabidopsis, and sugar beets and rice

We performed collinearity analysis within sugar beet itself and identified 18 pairs of collinear relationships (12 pairs of RING-H2, 4 pairs of RING-HCa, and 2 pairs of RING-v) (Fig. 3). The number of collinear pairs within sugar beet is relatively low, suggesting that sugar beet RING family members may have experienced few duplication events. We also conducted collinearity analysis of sugar beet RING members with other species. Sugar beet and its close relative spinach (both belonging to the order Caryophyllales) had 237 collinear pairs, indicating relatively high conservation in chromosomal arrangement. Sugar beet and the model plant Arabidopsis (both dicots) had 209 collinear pairs, suggesting strong overall conservation of the RING family members, although some members have undergone structural differentiation. Sugar beet and the distantly related crop rice (a monocot) had the lowest family conservation, with most members losing collinearity due to long-term evolution. We further calculated Ka/Ks ratios for the collinear pairs (Supplementary Fig. 2). The results showed that all Ka/Ks values were less than 1, with the majority (> 50%) being less than 0.3, indicating strong purifying selection and suggesting that this family is under functional constraint during evolution.

### Phylogenetic and conserved domain analysis of sugar beet RING-type proteins

**Fig. 4:**
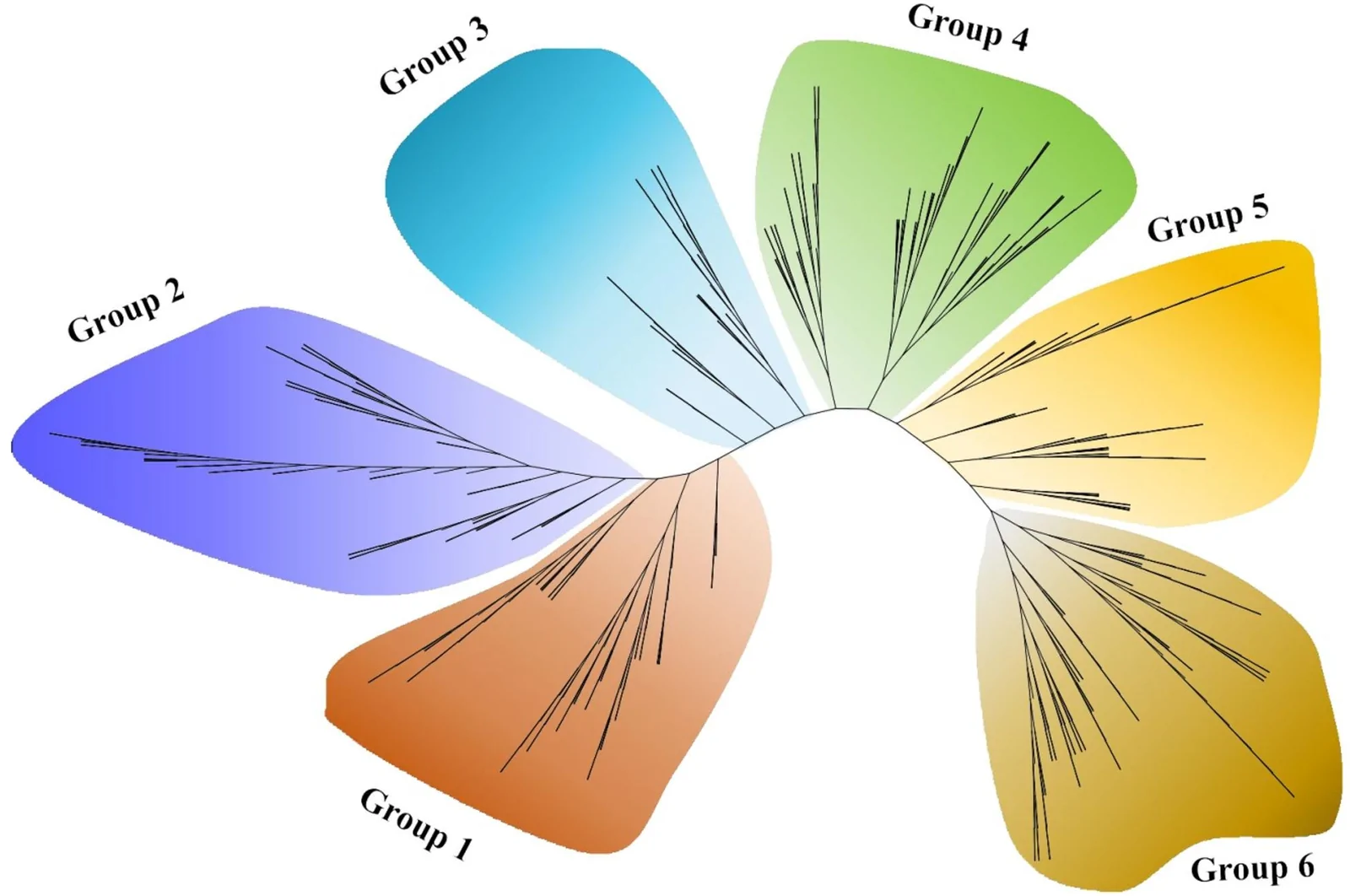
Phylogenetic tree of the RING-type family in sugar beet. The tree was constructed in MEGA software using the ClustalW function, with the maximum likelihood method and 1000 bootstrap replicates. Different background colors represent the different groups (1 to 6)

To explore the evolutionary relationships of the sugar beet RING family, we constructed a phylogenetic tree (Fig. 4). Due to the many branches in the phylogenetic tree, we manually divided it into six groups (the members in each group can be observed in Supplementary File 1. We also used tbTools to extract the physicochemical properties of all the protein members (including Molecular Weight, Theoretical pI, Instability Index, Aliphatic Index, and Grand Average of Hydropathicity) (Supplementary File 2) and analyzed the conserved domains and potential functions of the members. We obtained conserved domains for the 446 members using CD-search. After removing incomplete domains, members with similar conserved domains were found to cluster on the same branch. Majority of the members contain only the RING domain (306/446), which is the primary functional domain for ubiquitination. Other domains are mainly involved in processes of protein degradation (HRD/SPRY_RNF123), nucleic acid binding (RRM_SF/HIRAN), epigenetic regulation (SNF/SAD_SRA), complex assembly (vWA/ANKYR), enzymatic catalysis (PKc/RNase_H), signal transduction (GUB_WAK_bind/PLN03208), phosphorus metabolism (SPX_BAH1), cell division (CENP_E), and immune response (HepA/MLKL_NTD/Ubl1_cv_Nsp3) (Appendix Table 4). These domains are associated with various cellular processes, including protein degradation, nucleic acid binding, epigenetic regulation, and signal transduction, providing preliminary insights into the potential functional diversity of the sugar beet RING family.

### Analysis of *cis*-acting elements in the promoters of sugar beet RING-type genes

**Fig. 5:**
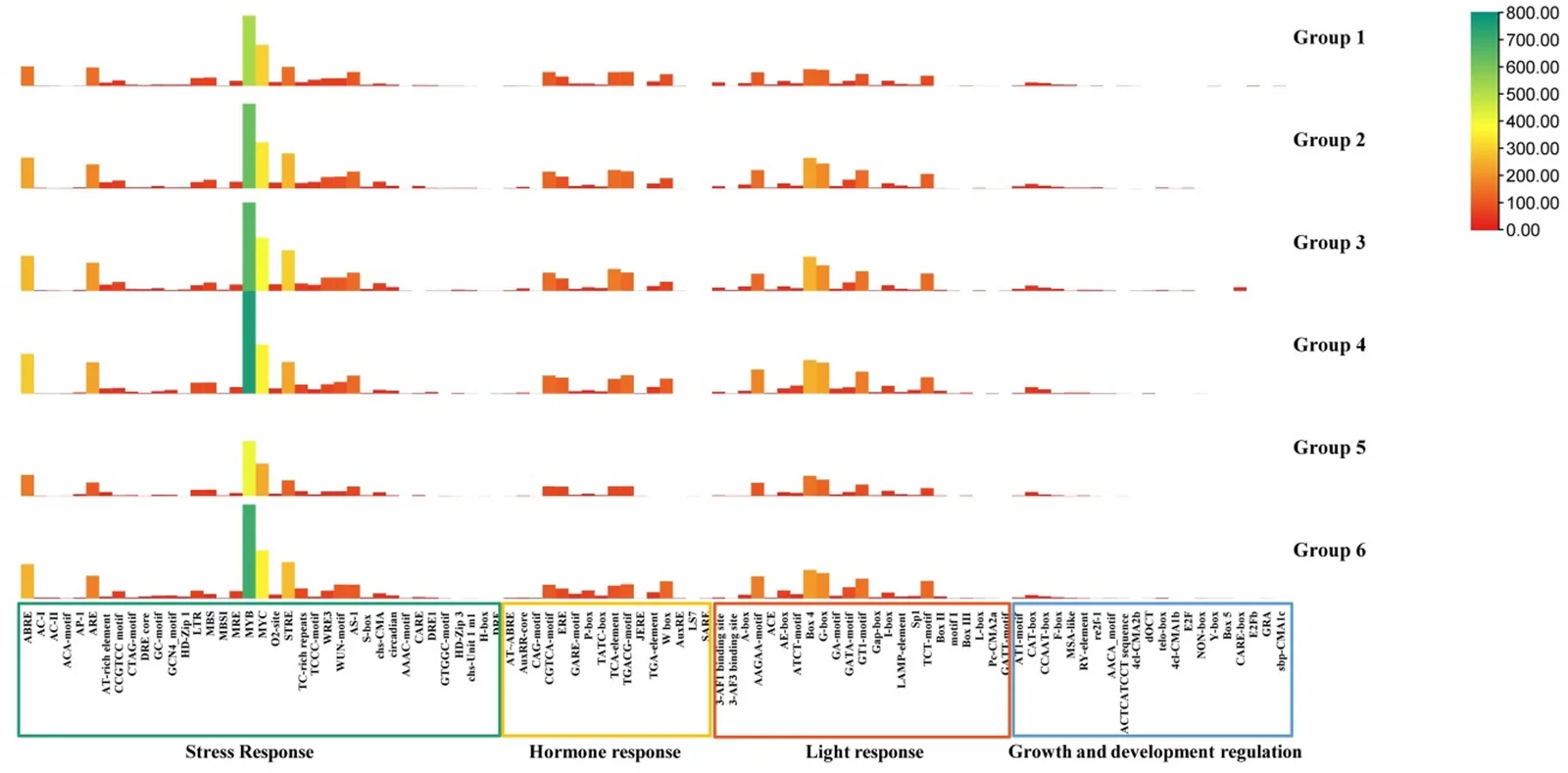
*Cis*-acting elements in the promoters of RING-type family members in sugar beet. Boxes of different colors represent distinct element types: green, stress-responsive elements; yellow, hormone-responsive elements; red, light-responsive elements; and blue, growth and development-related regulatory elements. The bar chart represents the relative count of each element type based on the color scale from 0 to 800

To determine potential transcriptional regulation of the sugar beet RING genes, we used the 2000 bp upstream of the coding genes as promoter sequences and analyzed their *cis*-acting elements. Results showed that in addition to *cis*-elements containing CAAT-box and TATA-box for transcription binding, four major categories of elements were revealed: stress response elements (37 types), hormone response elements (16 types), light response elements (22 types), and growth/development regulatory elements (21 types). Some elements like MYB and MYC have multiple functions, participating in both stress response and hormone signaling (Fig. 5). Overall, the *cis*-acting elements in the gene promoters are concentrated in stress response, mainly MYB, MYC, AREB, STRE, and ARE; among hormone response elements, they mainly focus on AT-ABRE, TCA-motif, and CGTCA-box, which are functional elements for abscisic acid (ABA), salicylic acid (SA), and methyl jasmonate (MeJA) responses, respectively. Box 4, G-box, and GT1-motif elements are the main light-responsive elements in the promoters. Growth and development regulatory elements were fewer in number than the other three categories, with CAT-box being the main element. The presence of diverse *cis*-acting elements, particularly those associated with stress, hormone, and light responses, suggests that the transcription of the RING family members may be regulated by multiple signaling pathways.

### Analysis of expression profiles of sugar beet RING-type genes under salt stress

**Fig. 6:**
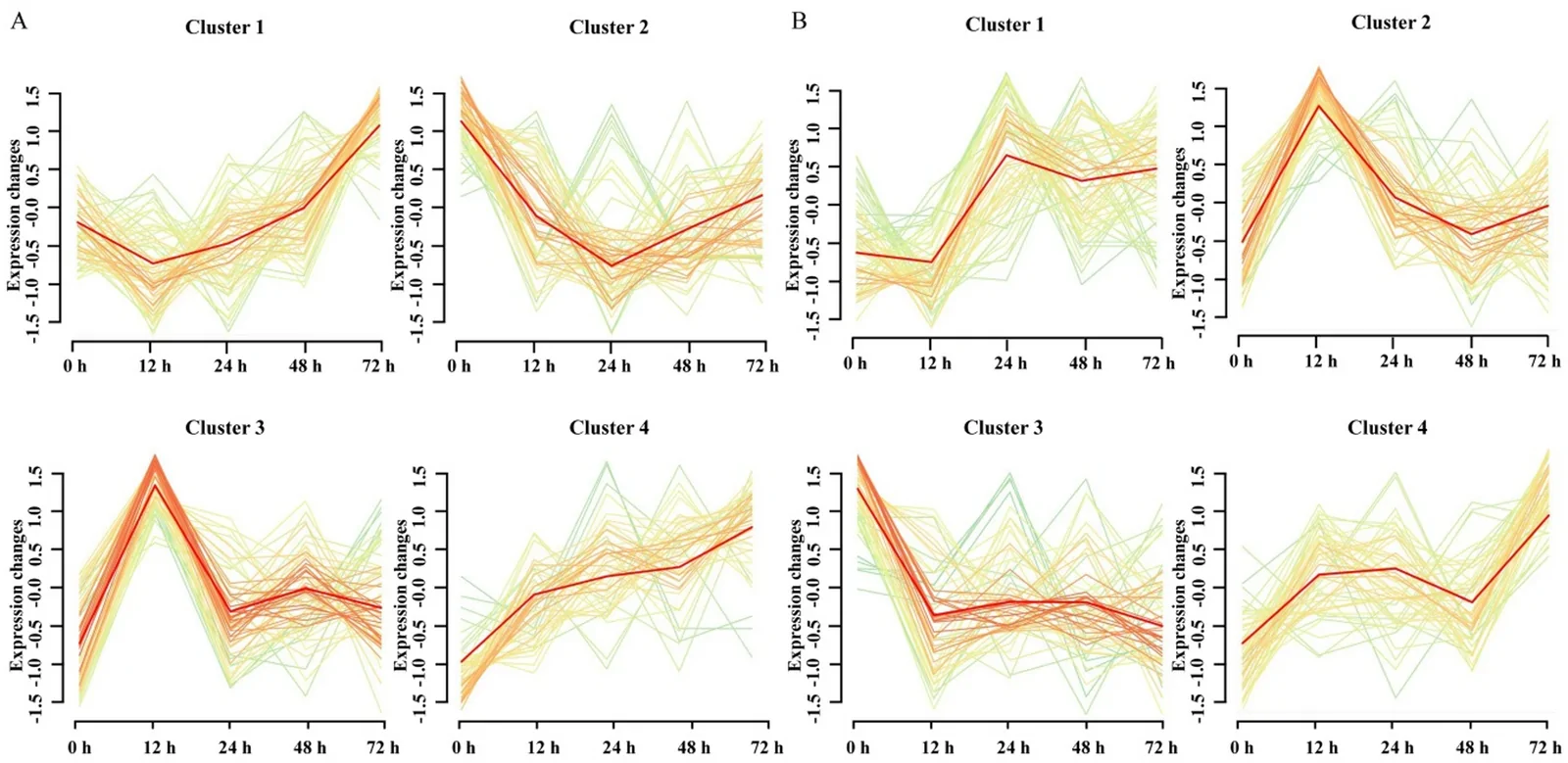
Clustering trend of *RING* gene expression profiles in sugar beet under salt stress. **A** Leaves; **B** Roots

Sugar beet exhibits relatively strong salt tolerance. Using transcriptomics data under salt stress, we analyzed the gene expression profiles of sugar beet RING family members in roots and leaves. Their expression patterns in leaves and roots under salt stress were clustered into four clusters each (Fig. 6). In leaves, cluster 1 showed fluctuation followed by a rise, reaching a peak only at 72 h; Cluster 2 decreased first then increased, reaching a trough at 24 h before slowly recovering; Cluster 3 sharply increased to a peak at 12 h, and subsequently dropped quickly and maintained a low level; Cluster 4 slowly increased, with only a slight recovery at 72 h. Overall, the expression of the 4 leaf clusters was relatively lagged and gentle. In roots, Cluster 2 sharply increased to a peak at 12 h (amplitude up to 2.5-fold), representing the earliest significant change among all clusters; Cluster 1 decreased first then stabilized, reaching a trough at 12 h before recovering quickly; Cluster 3 decreased amidst fluctuation, maintaining a low level after 12 h; Cluster 4 continuously increased, reaching a peak at 72 h. The key changes (increase/decrease) for most root genes were concentrated within 12–24 h, reflecting their early rapid response to salt stress. Roots are rapid-initiating, showing intense expression fluctuations within 12 h (e.g., the peak in Cluster 2), entering a stable/adjustment phase after 24 h; leaves are slow-adapting, with only Cluster 3 showing intense fluctuation at 12 h, and significant changes for most genes concentrated after 24 h. Among them, *BvRING-HCa-9* clustered into upregulated expression clusters in both roots and leaves (Cluster 1 and Cluster 2, respectively). Additionally, comparison of *BvRING-HCa-9* expression levels between the salt-sensitive sugar beet line S710 and the salt-tolerant line T710MU showed its expression was significantly elevated in the latter (Supplementary Fig. 4), suggesting that its potential contribution to salt tolerance.

### E3 ubiquitin ligase activity and subcellular localization assay

**Fig. 7:**
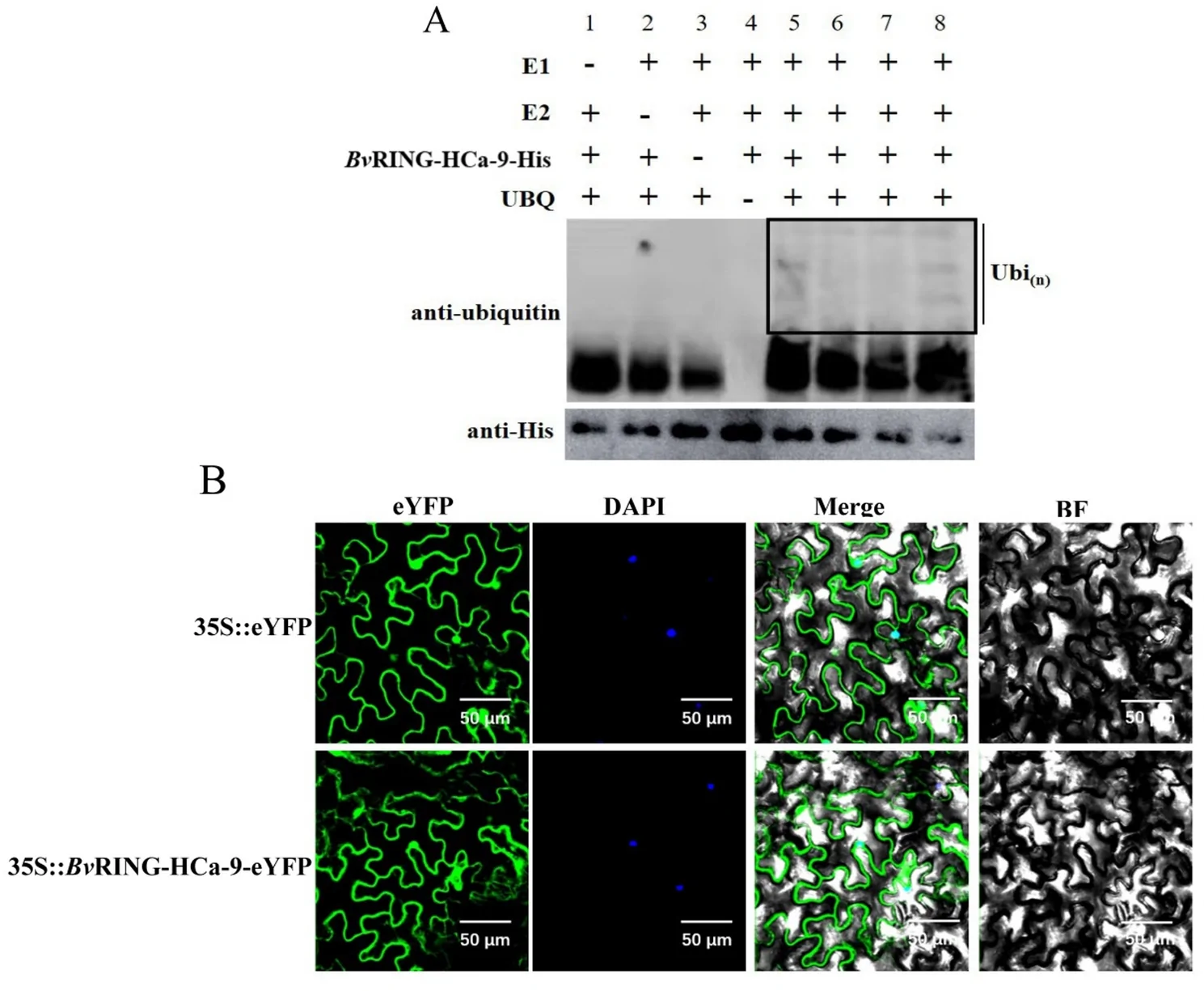
E3 ubiquitin ligase activity and subcellular localization of *BvRING-HCa-9*. **A** In vitro analysis of *BvRING-HCa-9* E3 ubiquitin ligase activity. The His-*BvRING-HCa-9* fusion protein was expressed in BL21 (DE3) strain and purified to determine its E3 ubiquitin activity in the presence or absence of E1, E2, or E3. The ubiquitinated protein bands were detected by using an anti-ubiquitin antibody; **B** Subcellular localization of *BvRING-HCa-9-*YFP fusion proteins transiently expressed in *N. benthamiana*. Bars = 50 μm

The sugar beet *BvRING-HCa-9* gene is significantly upregulated in the salt-tolerant sugar beet line T710MU, and the encoded protein consists of 186 amino acids. Western blot analysis was performed to assess the ubiquitin ligase activity of *Bv*RING-HCa-9 (Fig. 7A). Anti-His antibody was used to confirm equal loading of the eight protein combinations. Since the *Bv*RING-HCa-9 and UBCH5b are of similar MWs (differences of about 2 kDa), the anti-His band may represent both proteins. Anti-ubiquitin antibody was used to detect the ubiquitin ligase activity. To observe ubiquitin E3 ligase activity, the key is to observe a series of high MW bands in the Ubi(n) region, which indicate poly-ubiquitination on different target proteins. Lane 1 to 4 serve as negative controls, each missing a key component of the system. No poly-ubiquitinated protein bands were observed in any of the negative controls. In lane 3 where E2 was added, but not E3, there was no signal of poly-ubiquitination either, so the activity is not caused by E2. When all the components are present, especially with E3 (as shown in lanes 5 to 8), positive results of ubiquitination were observed. Both the nucleus and cell membrane showed fluorescent signals from the *Bv*RING-HCa-9-eYFP fusion protein, while in the Merge view, eYFP as a control showed fluorescent signals overlapping with the blue DAPI signal in the cytoplasm (Fig. 7B).

### Overexpression of sugar beet *BvRING-HCa-9* improves salt tolerance in Arabidopsis

**Fig. 8:**
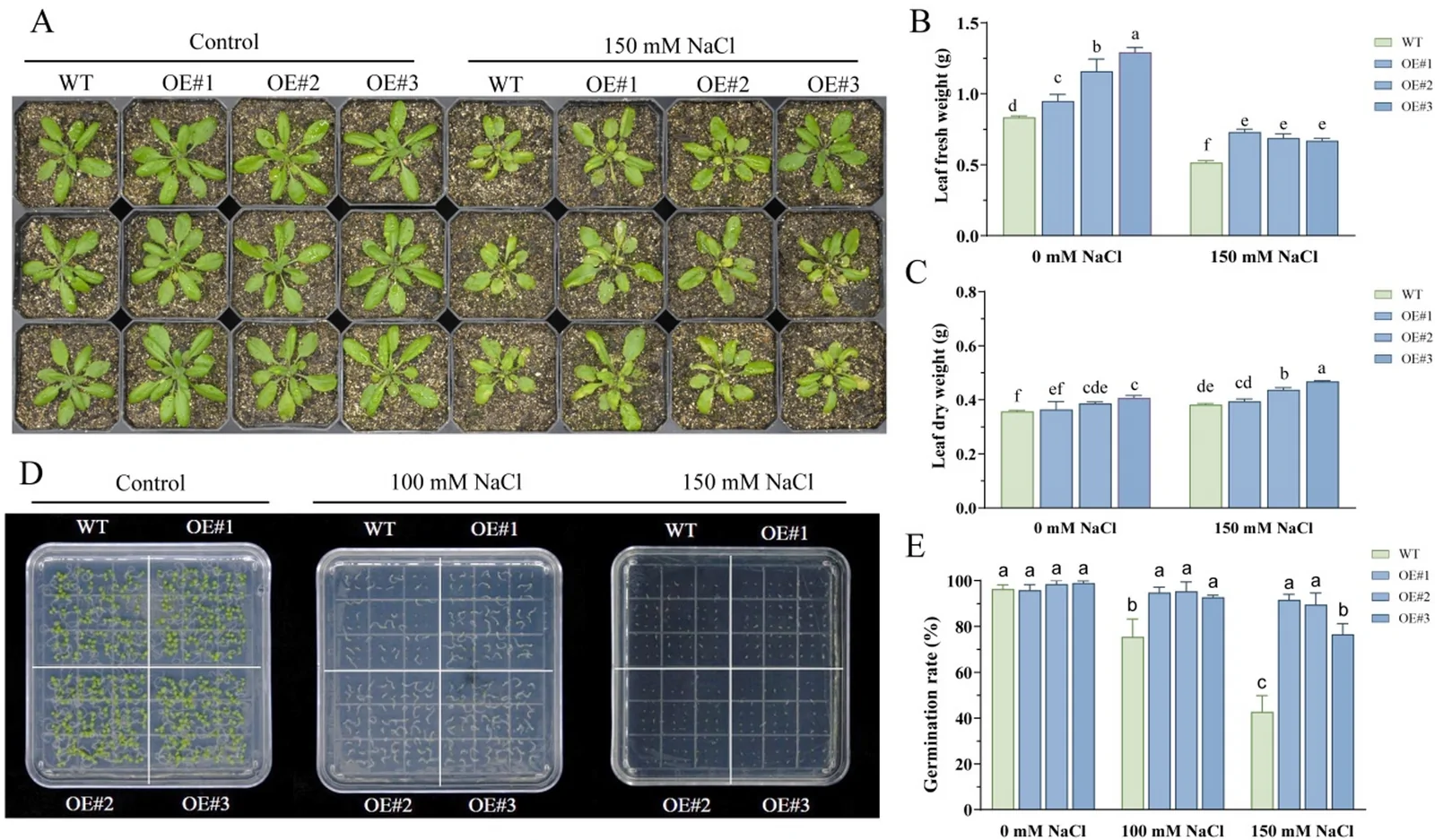
Salt stress tolerance of WT and *BvRING-HCa-9*-overexpressing Arabidopsis plants. **A** Phenotype of OE plants (OE#1, OE#2 and OE#3) and WT under 150mM NaCl; **B** Leaf fresh weight of WT and OE lines in (**A**); **C** Leaf dry weight of WT and OE lines in (**A**); **D** Germination rates of WT and OE lines under 100 mM and 150 mM NaCl treatments. Different letters on top of the bars in (**B**), **C**, and (**E**) indicate significant statistical differences (*p* < 0.05)

We obtained three stable *BvRING-HCa-9* overexpressing Arabidopsis lines (OE1, OE2, and OE3). First, we examined the phenotypes of WT and Arabidopsis lines before and after salt stress (Fig. 8A). Regarding leaf DW and FW, the OE plants were significantly heavier than WT plants under both salt stress and normal conditions (Fig. 8B and C). Also, under normal conditions, the seed germination rate of the transgenic plants showed no difference from WT plants, but after salt treatment, overexpression of the *BvRING-HCa-9* gene improved it, and cotyledon formation during seed germination was observed under 100 mM NaCl treatment (Fig. 8D). Based on these findings, overexpression of *BvRING-HCa-9* may improve the seed germination rate of Arabidopsis under 100 mM NaCl stress. Under 150 mM NaCl treatment, the root length of transgenic plants was significantly longer than that of WT plants, consistent with the germination rate results (Supplementary Fig. 5). In summary, the OE of *BvRING-HCa-9* promoted the growth of Arabidopsis plants and increased plant biomass, suggesting a role in salt stress tolerance.

**Fig. 9:**
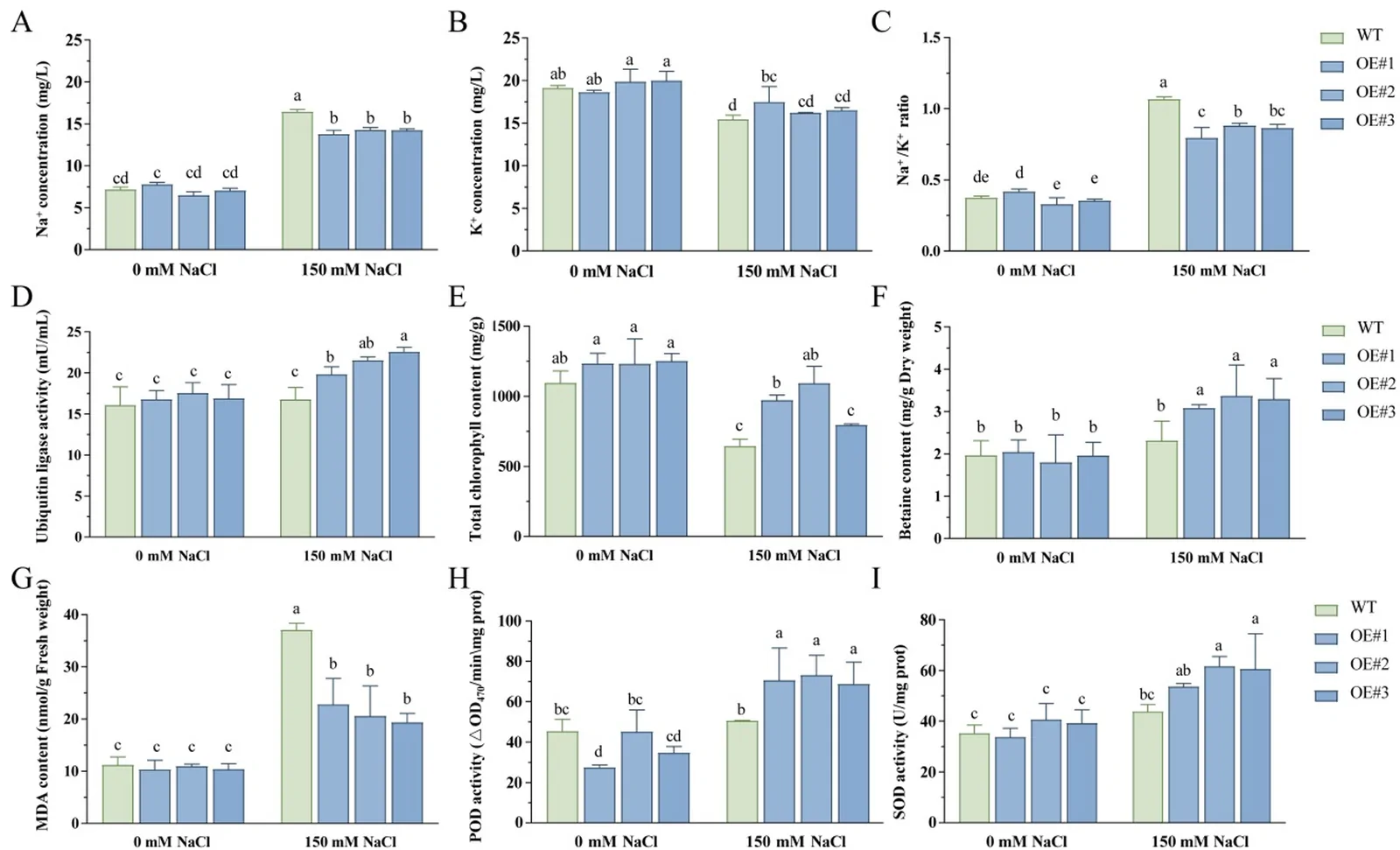
Physiological indexes of WT and *Bv*RING-HCa-9-overexpression plants under normal and 150 mM NaCl conditions. **A** Na^+^ concentration; **B** K^+^ concentration; **C** Na^+^/K^+^ ratio; **D** Ubiquitin ligase activity; **E** Total chlorophyll content; **F** Betaine content; **G** MDA content; **H** POD activity; **I** SOD activity. Data represent the mean ± standard deviation (SD) (*n* = 3). Different letters on top of the bars indicate significant statistical differences (*p* < 0.01)

Under normal conditions, there were no significant differences between WT and the OE plants in potassium ion (K^+^) content, sodium ion (Na^+^) content, or Na^+^/K^+^ ratio. However, after salt stress, the Na^+^ content and Na^+^/K^+^ ratio in the *BvRING-HCa-9* OE plants were significantly lower than in WT (Fig. 9A-C). Thus, OE of *BvRING-HCa-9* was associated with reduced Na⁺ accumulation and a lower Na⁺/K⁺ ratio under salt stress, suggesting improved ion homeostasis. The total E3 ubiquitin ligase activity of the plants was also measured. Results showed that only under salt stress conditions was the E3 ligase activity of the OE plants significantly higher than that of the WT (Fig. 9D). The photosynthetic capacity of the OE plants was evaluated. Under normal conditions, there was no significant difference in total chlorophyll content. After salt treatment, the content in the OE plants was significantly higher than in WT plants (Fig. 9E). Therefore, under salt stress, Arabidopsis plants expressing *BvRING-HCa-9* maintained higher total chlorophyll content, which may contribute to photosynthetic capacity. Before salt stress, betaine content showed no significant difference between the WT and OE plants. We found that after salt treatment, the betaine content in the OE plants was significantly higher than in WT plants (Fig. 9F). After salt stress, tissues or organs produce malondialdehyde (MDA) due to oxidation reactions, so MDA content can be measured to determine plant stress resistance levels. After salt treatment, MDA content in heterologously expressing plants was significantly lower than in WT (Fig. 9G). After salt stress, reactive oxygen species (ROS) content in Arabidopsis plants changes; therefore, the ability of plants to scavenge ROS was evaluated by measuring superoxide dismutase (SOD), and peroxidase (POD) activity in Arabidopsis plants overexpressing *BvRING-HCa-9* before and after salt stress. Under normal conditions, there was no significant difference in SOD and POD activity between the OE and WT plants. After salt treatment, SOD and POD activity in OE plants were significantly higher than in WT (Fig. 9H-I). Overall, OE of the sugar beet *BvRING-HCa-9* improved the salt stress tolerance of Arabidopsis at the physiological level.

### Changes in expression of salt stress-responsive genes in the *BvRING-HCa-9* transgenic Arabidopsis

**Fig. 10:**
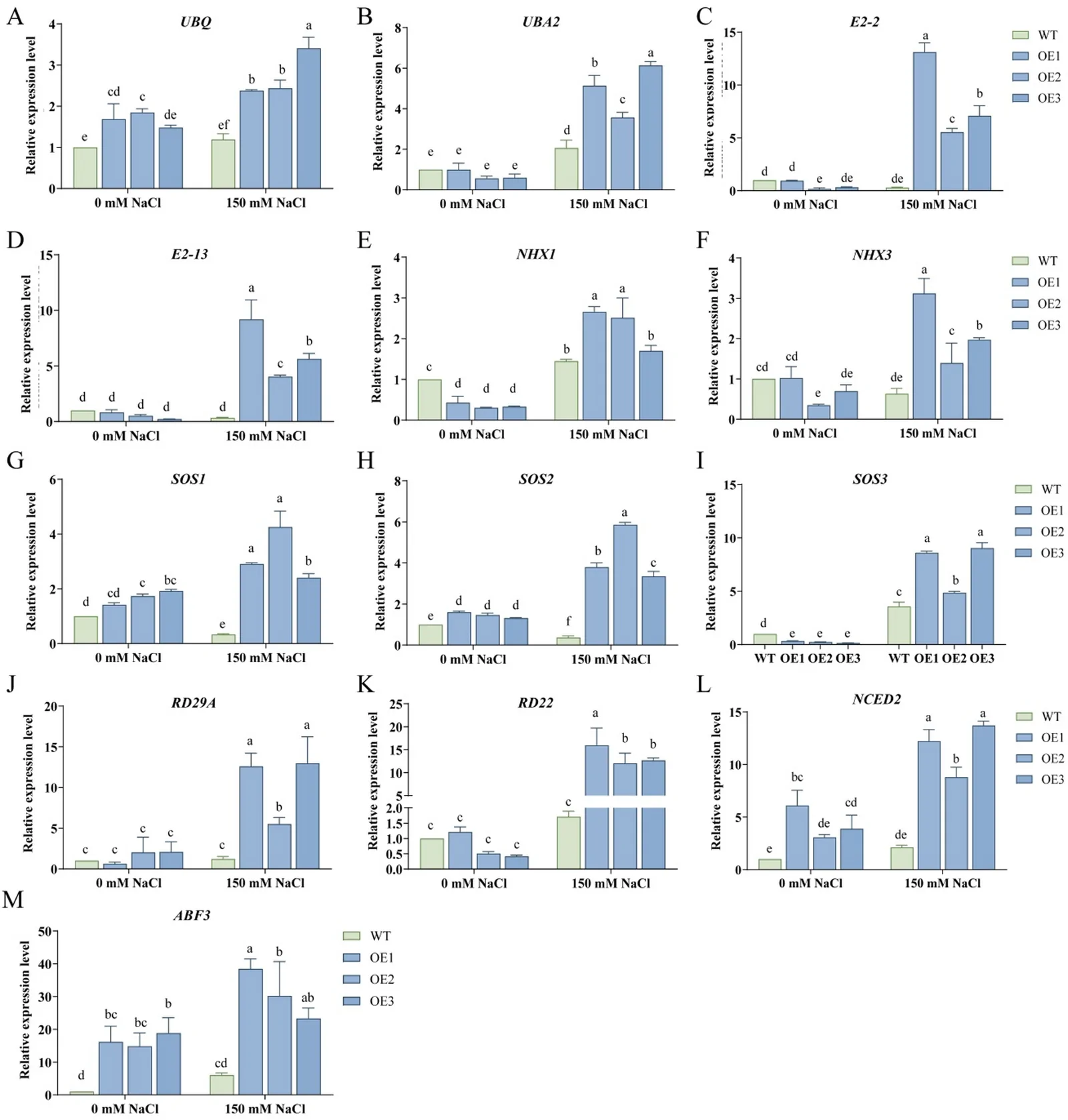
Gene expression analysis of WT and *Bv*RING-HCa-9 OE lines in response to 150 mM NaCl treatment. **A***Ubiquitin* (*UBQ*); **B***Ubiquitin Activating Enzyme 2* (*UBA2*); **C***Ubiquitin Conjugating Enzyme E2-2* (*E2-2*); **D***Ubiquitin Conjugating Enzyme E2-13* (*E2-13*); **E***Na⁺/H⁺ exchanger 1* (*NHX1*); **F***Na⁺/H⁺ exchanger 3* (*NHX3*); **G***Salt Overly Sensitive 1* (*SOS1*); **H***Salt Overly Sensitive 2* (*SOS2*); **I***Salt Overly Sensitive 3* (*SOS3*); **J***Responsive to Desiccation 29 A* (*RD29A*); **K***Responsive to Desiccation 22* (*RD22*); **L***9-Cis-Epoxycarotenoid Dioxygenase 3* (*NCED3*); **M***ABRE Binding Factor 3* (*ABF3*). The *Actin* gene was used as an internal reference. Data represent the mean ± standard deviation (SD) (*n* = 3), *P* ≤ 0.01

To explore the molecular mechanisms underlying the altered salt tolerance in the OE plants, we analyzed the expression levels of multiple genes using quantitative real-time PCR (qRT-PCR). First, the expression of ubiquitin system-related genes (including *AtUBQ*, *AtUBA2*, *AtE2-2*, and *AtE2-13*) was detected (Fig. 10A-D). The expression of the ubiquitin gene (UBQ) in OE plants was significantly higher than in WT, and this trend was consistent before and after salt treatment, indicating that heterologous expression of *BvRING-HCa-9* was associated with increased transcript levels of ubiquitin genes. Before salt stress, there was no significant difference in expression of the ubiquitin-activating enzyme gene *AtUBA2*, and the ubiquitin-conjugating enzyme genes *AtE2-2* and *AtE2-13* between the WT and OE plants; whereas after salt stress, the expression of these genes in OE plants was significantly higher than in WT, suggesting that the higher transcript levels of ubiquitin-activating enzyme and ubiquitin-conjugating enzyme genes under salt stress may contribute to enhanced plant salt tolerance, potentially via increased ubiquitination modification.

Secondly, the expression of typical salt stress-responsive genes and Salt Overly Sensitive (SOS) pathway-related genes (such as *AtRD22*, *AtSOS1*, etc.) was quantified (Fig. 10E-M). Before salt treatment, there was no significant difference in expression of salt-responsive genes *AtRD22*, *AtRD29A*, and *AtSOS2* between WT and OE plants, but SOS1 gene expression in OE plants was significantly higher than WT, while *SOS3* gene expression was significantly lower than WT; after salt stress, the expression of all detected genes in OE plants was significantly higher than in WT. It was also found that after salt stress, the expression levels of *AtNHX1* and *AtNHX3* genes in OE plants were significantly upregulated. Furthermore, the expression of the key ABA synthesis gene *AtNCED2* and the gene encoding an important transcription factor in the ABA signaling pathway, AtABF3, was detected: before and after salt treatment, the expression of *AtNCED3* and *AtABF3* in the OE plants was significantly higher than in WT; before salt stress, *AtABI3* gene expression in the OE plants was significantly higher than WT, but after salt treatment, there was no significant difference; whereas *AtABI5* gene expression in OE plants was significantly lower than WT both before and after salt treatment (Fig. 10L-M). These results showed that OE of *BvRING-HCa-9* was associated with altered expression of multiple genes involved in the ubiquitin system, salt stress response, SOS pathway, and ABA signaling. Under salt stress, OE plants exhibited higher transcript levels of most detected genes, including salt-responsive markers (*AtRD22*, *AtRD29A*), ion transporters (*AtSOS1*, *AtNHX1*, *AtNHX3*), and ABA-related genes (*AtNCED3*, *AtABF3*). These expression patterns are consistent with the improved physiological performance and salt tolerance observed in *BvRING-HCa-9* OE plants.

## Discussion

### Evolutionary Conservation of RING-type Proteins in Sugar Beet

The sugar beet *Bv*RING family contains 446 members, which are distributed across all nine chromosomes, with varying subtype composition among chromosomes. Its number is fewer than flax (574) and tomato (469), but more than the monocot rice (425). However, the number of RING-type proteins is similar across different species [32, 33]. Sugar beet has few collinear pairs with itself, indicating that only a small portion of its members were produced by duplication events, which also aligns with the characteristics of RING family members in most species (cotton, wheat, potato) [34]. The results of collinear pair numbers between sugar beet and spinach, Arabidopsis, and rice show that the evolutionary conservation of the RING gene family is positively correlated with the closeness of species kinship, and exhibits the characteristic of Ka/Ks < 1 (mostly < 0.3), indicating the family has undergone extremely strong purifying selection during species differentiation, maintaining functional conservation. Therefore, the 446 members of the sugar beet RING family likely arose primarily through gradual differentiation during species evolution, with limited contributions from recent duplication events [35, 36].

The sugar beet RING family is classified into nine categories based on the position and composition of the eight metal ligands in the conserved domain. Among them, RING-H2 (48.7%) and RING-HCa (34.8%) categories have a significant numerical advantage, similar to trends in plants like maize, poplar, and flax [37]. This result suggests that these two domains are evolutionarily conserved and may represent the core functional components of the RING family in eukaryotes, as well as the major contributors to the numerical expansion of this family in plants [38]. The amino acid spacing between adjacent ligands is a core factor affecting the structural stability, conformational plasticity, and functional specificity of metal-binding proteins [39, 40]. Most ligand pair spacings in sugar beet RING proteins are stable, e.g., ml1-ml2 is mostly 2, ml3-ml4 is mainly 1, ml7-ml8 is mainly 2, with only ml2-ml3 and ml6-ml7 being the main variable regions. The ml3-ml4 spacing in RING-v is concentrated at 7. The difference of 2 vs. 4 amino acids between the ml7-ml8 ligands distinguishes the structure of RING-HCa and RING-HCb. Unlike other types, RING-H2 has ml2-ml3 mainly at 14/15 amino acids and ml6-ml7 mainly at 10; RING-HCa has the two spacings enriched at 11 and 10 respectively, while RING-HCb is concentrated at 13 and 19 respectively. The RING domain relies on cysteine-rich motifs to chelate two zinc ions, and ligand spacing is key to maintaining the conformational stability of zinc ion binding sites. Stable site spacing is a necessary condition for E3 activity [41]. RNF4 contains a RING conserved domain; altering ligand spacing causes conformational disorder at the zinc ion binding sites of RNF4, and Arabidopsis RNF4 cannot correctly position the E2-ubiquitin complex, thereby losing its E3 activity to catalyze K63-linked ubiquitin chain synthesis [42]. However, the specific impact of these differences in amino acid spacing on zinc ion binding ability and protein function requires more experimental evidence.

### Involvement of RING proteins in multiple biological processes

All the sugar beet *Bv*RING family members contain the conserved RING domain, while the numbers of other domains vary, corresponding to diversified functions. Members containing only the single RING domain (306) are the core group of the family. These proteins primarily mediate ubiquitination through the RING domain, participating in basic physiological processes like protein degradation [43]. For example, RIE1 (RING-finger Protein for Embryogenesis 1) is an E3 ubiquitin ligase containing only the single RING domain. It can bind and mediate the ubiquitination of the ethylene synthesis rate-limiting enzyme *At*ACS7 (1-Aminocyclopropane-1-Carboxylate Synthase 7), regulating ethylene synthesis levels. When RIE1 function is lost, *At*ACS7 accumulates, ethylene content increases, and plant leaf senescence accelerates [44]. Wheat *Ta*AIRP2-1B (ABA-insensitive RING Protein 2-1B) is an E3 ubiquitin ligase gene containing a RING-HC type domain. It functions by interacting with *Ta*HIPP3 (Heavy Metal-Associated Isoprenylated Protein 3) to promote its degradation, positively regulating wheat spike length [45]. *At*ARRE (Arabidopsis RING-type E3 ligase regulating ECERIFERUM), another E3 ubiquitin ligase containing a RING domain in Arabidopsis, targets key wax synthesis enzymes CER1 (Eceriferum 1) and CER3 for degradation via the ubiquitination pathway, reducing wax accumulation on stems and leaves. It enhances plant resistance to pathogens by optimizing wax composition, and regulating plant growth and defense against pathogen invasion [46].

When RING-type proteins contain other domains, they exhibit more complex functions. Arabidopsis *At*NLA1 (Nitrogen Limitation Adaptation 1) contains RING and SPX domains. It can ubiquitinate and degrade phosphate transporters *At*PHT1s (Phosphate Transporter 1). Similarly, rice *Os*NLA1 degrades phosphate transporters like *Os*PT2 and *Os*PT8, thereby regulating phosphorus uptake and homeostasis [47]. Arabidopsis BTS protein contains 3 HHE domains and 1 RING domain. The HHE domains bind iron ions and regulate its own stability. The RING domain relies on its E3 ligase activity to interact with PYEL (POPEYE-Like) class transcription factors in the nucleus to mediate their 26 S proteasomal degradation, thereby regulating iron deficiency response-related gene expression. Meanwhile, BTS (BRUTUS) participates in regulating root growth, rhizosphere acidification, and iron reductase activity, being crucial for plant growth, development, and iron uptake balance [48]. Apple RAD5B (RAD5 Homolog B) protein belongs to the category containing DEXDc and RING domains. Under drought stress, *Md*RAD5B interacts with *Md*LHP1 (Like Heterochromatin Protein 1), a key regulator of H3K27me3, and mediates its degradation. *Md*RAD5B affects the expression of stress-related genes through *Md*LHP1-mediated H3K27me3 deposition on drought-responsive genes, acting as a positive regulator of drought tolerance [49]. These examples from other plant species suggest that sugar beet RING-type E3 ubiquitin ligases are likely involved in similar processes, e.g., growth, development, nutrient transport, and stress responses, through the synergistic action of their functional domains.

### Extensive involvement of sugar beet *RING* genes in the salt stress response

Through transcriptomic analysis of the expression profiles of the 446 RING protein-coding genes in roots and leaves in response to salt stress, we observed that the overall RING gene response in roots was faster, with peaks appearing within 12 h of salt stress, entering a stable/adjustment phase after 24 h. In contrast, the overall RING gene changes in leaves after salt stress were concentrated after 24 h. This difference may reflect the distinct functional roles of roots and leaves in salt stress responses. Directly exposed to salt stress, roots often initiate rapid transcriptional changes to activate tolerance mechanisms such as ion compartmentalization [50]. In contrast, leaves, as photosynthetic organs, may adopt a more delayed response, possibly to sustain photosynthetic function under stress [51, 52]. The expression patterns observed here suggest that many sugar beet *RING* genes are transcriptionally responsive to salt stress and may contribute to salt tolerance. Currently, functional studies on RING-type E3 ubiquitin ligase genes in sugar beet salt tolerance remain limited. Among the salt-responsive *RING* genes identified, *BvRING-HCa-9* was significantly upregulated in salt-tolerant varieties, making it an obvious candidate for further functional characterization.

### The *BvRING-HCa-9* confers salt tolerance on transgenic Arabidopsis thaliana

E3 ubiquitin ligases are important components of the ubiquitination system, mediating plant responses to abiotic stress [53]. COP1 (Constitutive Photomorphogenic 1) is a RING-type E3 ubiquitin ligase. Salt stress induces Ca²⁺ signaling, causing COP1 to translocate from the nucleus to the cytoplasm. In the cytoplasm, COP1 mediates 26 S proteasome-dependent degradation of the GI (GIGANTEA) protein, relieving GI’s inhibition of SOS2, thereby activating SOS1-mediated Na⁺ efflux and improving Arabidopsis salt tolerance [54] Under salt stress, Arabidopsis RING finger-type E3 ubiquitin ligases PIR1 (PP2CA Interacting RING Finger Protein 1) and PIR2 ubiquitinate and degrade the ABA signaling negative regulator PP2CA (Protein Phosphatase 2 C A), relieving its inhibition on SnRK2 (Sucrose non-fermenting 1-Related Protein Kinase 2) kinases, enhancing ABA pathway response. This mechanism can promote stomatal closure and reduce water loss, improving plant salt tolerance [55]. The RING-type E3 ubiquitin ligase *Sl*MIEL1 (MYB30-Interacting E3 Ligase 1) is localized in the nucleus. Under salt stress, *Sl*MIEL1 ubiquitinates and degrades *Sl*NAC35, relieving inhibition on *Sl*AOC, promoting JA accumulation, further activating SOD and CAT activity, efficiently scavenging excess ROS and reducing membrane damage, thereby enhancing tomato salt tolerance [56]. Additionally, *Ta*GW2 (Grain Width 2), a negative regulator of salt tolerance in wheat, ubiquitinates the positive regulator *Ta*VOZ1 (Vascular plant One-ZINC Finger 1) to promote its degradation. In addition to target protein degradation, *Ta*GW2 acts as a transcriptional repressor that competes with *Ta*VOZ1 for binding to the *HKT1* promoter, thereby inhibiting *HKT1* transcription and reducing salt tolerance [57]. In this study, *Bv*RING-HCa-9 was localized to the nucleus and plasma membrane. By measuring physiological parameters, transcription changes, and phenotypes of OE plants under salt stress, we found that OE of the *BvRING-HCa-9* was associated with increased transcript levels of selected *E1* and *E2* genes, elevated total E3 ligase activity, and altered expression of genes involved in the SOS pathway, ABA signaling, and osmotic adjustment. Consistent with these molecular changes, transgenic plants exhibited lower Na⁺ accumulation, higher antioxidant enzyme activity, and improved growth performance under salt stress. These observations suggest that *BvRING-HCa-9* may contribute to salt tolerance through multiple coordinated effects on ion homeostasis, osmotic balance, and oxidative stress mitigation. However, although *Bv*RING-HCa-9 possesses E3 ubiquitin ligase activity, whether it promotes salt tolerance through ubiquitination of target proteins remains to be determined. 

## Conclusion

The identified 446 *Bv*RING genes in the sugar beet genome were distributed across all nine chromosomes with varying subtype abundance. The RING family is evolutionarily conserved across angiosperms, with limited duplication events after species divergence. Conserved domain analysis revealed structural diversity among *Bv*RING members, and many of the genes are transcriptionally responsive to salt stress. Among them, *Bv*RING-HCa-9 was identified as a candidate involved in salt tolerance, as it exhibited E3 ligase activity, localized to the nucleus and plasma membrane, and was upregulated in salt-tolerant sugar beet varieties. Overexpression of *BvRING-HCa-9* in Arabidopsis enhanced salt tolerance, which was associated with reduced Na⁺ accumulation, increased antioxidant enzyme activity, and improved growth performance. Thus, *BvRING-HCa-9* represents a promising candidate for further functional studies and potential application in molecular breeding for salt tolerance. The other *Bv*RING genes identified in this study provide a valuable resource for future functional characterization of RING-type E3 ligases in plant stress responses.

## Supplementary Information

Supplementary Material 1.

Supplementary Material 2.

Supplementary Material 3.

Supplementary Material 4.

Supplementary Material 5.

Supplementary Material 6.

Supplementary Material 7.

Supplementary Material 8.

Supplementary Material 9.

Supplementary Material 10.

Supplementary Material 11.

## Data Availability

The datasets used and/or analysed during the current study are available from the corresponding author on reasonable request.
